# Feline Hemotropic *Mycoplasma* Species of Apparently Healthy Domestic Cats in Konya Province of Türkiye

**DOI:** 10.3390/vetsci11110530

**Published:** 2024-10-31

**Authors:** Ceylan Ceylan, Muhammed Hudai Culha, Gonca Sonmez, Muhammed Ahmed Selcuk, Merve Ider, Ayşe Evci, Sule Yılmaz, Ferda Sevinc, Onur Ceylan

**Affiliations:** 1 Department of Parasitology, Faculty of Veterinary Medicine, Siirt University, Siirt 56000, Türkiye; ceylan.ceylan@siirt.edu.tr (C.C.); ahmed.selcuk@siirt.edu.tr (M.A.S.); 2 Department of Genetics, Faculty of Veterinary Medicine, Selcuk University, Konya 42130, Türkiye; mhudai.culha@selcuk.edu.tr (M.H.C.); goncasen@selcuk.edu.tr (G.S.); 3 Department of Internal Medicine, Faculty of Veterinary Medicine, Selcuk University, Konya 42130, Türkiye; m.ider@selcuk.edu.tr; 4 Department of Parasitology, Faculty of Veterinary Medicine, Selcuk University, Konya 42130, Türkiye; ayse.evci@selcuk.edu.tr; 5 Health Science Institute, Selcuk University, Konya 42130, Türkiye; 193147002001@lisansustu.selcuk.edu.tr

**Keywords:** cat, hemoplasma, molecular detection, *Mycoplasma* spp., *Mycoplasma hemocanis*

## Abstract

Feline hemotropic mycoplasmosis is an emerging infectious disease in domestic cats caused by a group of hemotropic *Mycoplasma* species, including three main species: *Mycoplasma hemofelis* (*Mhf*), *Candidatus* Mycoplasma hemominutum (*C*Mhm), and *Candidatus* Mycoplasma turicensis (*C*Mt). The primary objective of this investigation was to ascertain the molecular prevalence and characterization of hemotropic mycoplasmas in domestic cats from twenty-three districts of Konya province in Türkiye. For this purpose, 384 apparently healthy cats belonging to different breeds were enrolled in this study. The molecular prevalence of *Mycoplasma* spp. in cats was 9.4%, and the identified species included *Mhf*, *Mycoplasma hemocanis* (*Mhc*), *C*Mt, and *C*Mhm. The molecular prevalence was not significantly associated with district, breed, or gender (*p* > 0.05) but was significantly associated with age and ownership status (*p* < 0.05). The study found that all cats with *Mycoplasma* spp. were older than one year and that the infection had a higher prevalence in stray cats than in owned cats (*p* < 0.05). Although there are studies conducted in different provinces, data on the molecular and phylogenetic characterization of the species causing feline hemotropic mycoplasmosis are scarce in Türkiye. This study, which provides updated data concerning the molecular characterization and phylogeny of hemotropic *Mycoplasma* species of cats in Türkiye, revealed the *Mycoplasma* spp. molecular prevalence in Konya province for the first time and provided remarkable findings that will fill the scientific gap in the country. The molecular characterization of these hemotropic mycoplasmas is crucial for understanding their epidemiology and developing effective diagnostic and treatment strategies for feline hemotropic mycoplasmosis. It is anticipated that the data from the study will raise awareness among pet owners, veterinarians, and healthcare professionals of feline hemotropic *Mycoplasma* agents with zoonotic potential.

## Simple Summary

This study investigated the presence of hemotropic *Mycoplasma* species, which cause an emerging disease in domestic cats, in Konya province, Türkiye. We tested 384 apparently healthy cats and found that 9.4% of them were infected with *Mycoplasma* species, including *Mycoplasma hemofelis*, *Mycoplasma hemocanis*, *Candidatus* Mycoplasma turicensis, and *Candidatus* Mycoplasma hemominutum. The prevalence of the infection was linked to the cats’ age and ownership status, with older and stray cats more likely to be infected. There was no significant association between infection rates and the districts, breeds, or genders of the cats. This study is the first to provide data on the molecular prevalence of these bacteria in Konya province and highlights the importance of understanding these infections to improve diagnostics and treatments. The findings also raise awareness about the potential zoonotic risk of these infections for humans.

## 1. Introduction

In recent years, emerging pathogens and parasitic zoonoses that threaten animal health and public health have attracted increasing attention, and feline vector-borne pathogens (FVBPs) have a significant place among them [1,2,3]. The growing trend of cat population in Türkiye and the increasing rate of cat ownership underline the importance of feline vector-borne diseases (FVBDs) and it is of great importance to diagnose and treat these diseases, to identify the carriers, and to implement the necessary control measures [4,5,6]. Cats are known to harbor a diverse array of arthropod-transmitted bacterial, protozoan, viral, and other parasitic blood pathogens, some of which are of zoonotic concern, although they are an integral component of human life [3]. However, the presence of many life-threatening zoonotic feline pathogens and research in this area has not been in step with the resurgence of interest in FVBDs, and lacking knowledge about FVBDs, in particular, severely hampers the development and implementation of effective control preventions at the regional and national scale [7].

Feline hemotropic mycoplasmosis is one of the most critical FVBDs and causes a clinical picture characterized by hemolytic anemia in cats [8,9]. Although the natural route of transmission of feline hemoplasmas among cats has not been fully elucidated, it is thought that arthropod vectors, mainly fleas and ticks, and less frequently direct cat-to-cat contact between cats, blood transfusion, and vertical transmission, may play a role in the spread of feline hemotropic mycoplasmosis [8]. Among the vectors, the cat flea *Ctenocephalides felis* is widely accepted as the most prominent vector for feline hemotropic mycoplasmosis [10]. The cat flea, which is frequently encountered in cats in Türkiye [11], is estimated to be the most significant ectoparasite in the transmission cycle of hemoplasma among cats in the country.

Many different methods are used to diagnose the infection. On a complete blood count, the most characteristic abnormalities seen in infected cats are macrocytosis, anisocytosis, reticulocytosis, polychromasia, Howell–Jolly bodies, and regenerative anemia with normoblastemia, which is sometimes pronounced, especially in cats [8,9,12]. Since hemoplasmas separate from erythrocytes within hours, blood smears should be prepared from fresh blood. Staining should be performed with properly prepared and non-contaminated dye solutions (Romanowsky type), as dye residues can lead to false-positive results at the point of diagnosis [9]. Currently, PCR-based methods applied to vector-borne pathogens, including hemotropic mycoplasmas, are very effective in detecting and characterizing microorganisms involved in the etiology of diseases, monitoring recovery after chemotherapy, and assessing the role that subclinically infected animals may play in the propagation of infections [13,14]. In parallel with the diagnostic developments in the world, PCR-based techniques have started to be used in Türkiye for the diagnosis of feline hemotropic mycoplasmosis, which was investigated cytologically until the 2000s [6,15,16,17]. The fact that hemotropic mycoplasmosis can be fatal in domestic cats in Türkiye [18,19], the high molecular prevalence in cats with suspicion of the disease [20,21], the prevalence of cat flea and the zoonotic potential of hemoplasmosis transmitted by these ectoparasites have increased interest in the subject [22]. Taken collectively, the existing literature points out a paucity of data about the molecular epidemiology and phylogeny of hemotropic mycoplasmas in domestic cats (*Felis catus*) from Türkiye. This study was designed to molecularly identify and characterize the *Mycoplasma* species involved in the etiology of hemotropic mycoplasmosis in cats from the Konya province, which is located in the Central Anatolian region of Türkiye and the center of the country.

## 2. Materials and Methods

### 2.1. Sampling Location and Collection of Blood Samples from Cats

**Figure 1:**
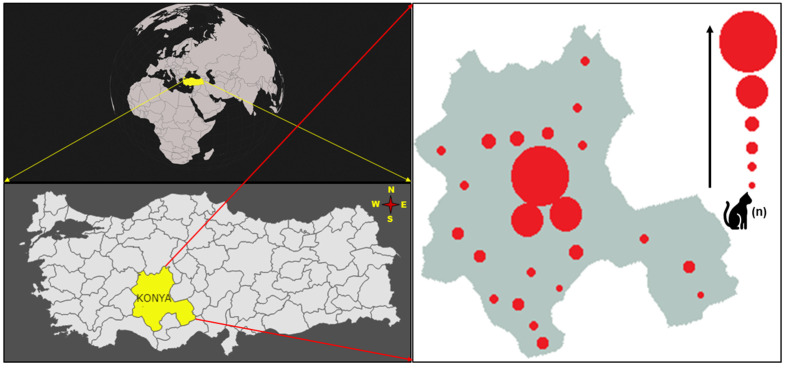
Konya districts where cat blood samples were sampled.

With a population of approximately 2.3 million, Konya is the largest province (40,838 km^2^) within the borders of the Central Anatolian Region of Türkiye and has 31 districts. It is noteworthy that the cat population in Türkiye, including Konya province, is increasing due to the adoption of cats around the world and the acceptance of cats as family members. Animal registrations to Selcuk University Veterinary Faculty Small Animal Hospital in Konya, one of the largest university hospitals in the region, also confirm this situation. Sample size calculation for cats included in the study was performed according to the formula *n* = [(1.96)^2^. *P_exp_*(1 − *P_exp_*)]/*d*^2^ described by Thrusfield [23]. *P_exp_*, *n*, and *d* represent the expected prevalence, the required sample size, and the desired absolute precision, respectively. In the absence of data regarding the *Mycoplasma* spp. prevalence in the study population, *P_exp_* was set at 50%, *d* at 5%, and the minimum number of cats that could be used in the study was set at 384. Within the scope of the study, 384 blood specimens were taken from cats admitted to the Small Animal Hospital of Selcuk University Veterinary Faculty for various purposes such as vaccination, antiparasitic application, sterilization, and routine control from different districts of Konya, especially Selçuklu, Meram, and Karatay between January 2020 and December 2021. All cats constituting the study material were apparently healthy by physical and morphological examinations. A small quantity of blood, ranging from 1 to 2 mL, was carefully drawn from the *Vena cephalica antebrachium* of each cat involved in the study and was taken into anticoagulant tubes (EDTA) with the help of a suitable cannula. The information about the cats, such as breed, age, gender, owner/stray cat status, and the districts where the cats were sampled, was checked, and necessary information was recorded. No ectoparasite infestations in cats have been recorded. The distribution of the collected samples by districts and the descriptive information regarding the sampled cats are shown in Figure 1 and Table 1, respectively.

### 2.2. Preparation of Thin Blood Smear

The collected blood samples were brought to the Department of Veterinary Parasitology, Faculty of Veterinary Medicine, on the same day, and thin blood smears were prepared at least two from each sample. The smears were dried and fixed with absolute methyl alcohol (Sigma-Aldrich, Saint Louis, MO, USA) for 5 min. The fixed blood smears were stained with 10% Giemsa stain (Merck, Darmstadt, Germany) for 45 min and were washed under tap water at the end of the time.

### 2.3. Separation of Plasma and Blood

Blood samples were centrifuged at 3000× *g* for 15 min to separate plasma and blood cells. Plasma and blood were placed in different Eppendorf tubes, and the samples were kept at −80 °C until genomic DNA isolation.

### 2.4. Genomic DNA Isolation

Genomic DNA (gDNA) isolation from blood samples was performed using a commercial DNA isolation kit (QIAamp^®^ DNA Blood Mini Kit, QIAGEN, Hilden, Germany). The obtained DNA samples were stored at −20 °C until used in molecular analyses.

### 2.5. Molecular Detection of Mycoplasma Spp.

The isolated gDNAs were analyzed using universal *Mycoplasma* spp. primers (HBT-F/R) to detect feline hemoplasmas (Table 2). A total of 50 µL of PCR mixture was prepared by complementing with 5X MyTaq Reaction Buffer, 20 µM primer, 1 µL MyTaq DNA Polymerase (Meridian Bioscience, Cincinnati, OH, USA), ddH_2_O to contain approximately 1 µg genomic DNA. PCR cycles were performed at 95 °C for 5 min, 40 cycles (95 °C 30 s, 57.2 °C 30 s, and 72 °C 1 min), followed by 72 °C for 15 min. Double-distilled water (Invitrogen, Ultrapure^TM^) and genomic DNAs from our previous study [6], which was confirmed to be *Candidatus* Mycoplasma hemominutum by sequencing (OR979179, OR979180), were used as negative and positive controls, respectively. PCR products were electrophoresed on 1.5% agarose gel and visualized in a UV transimulator (UVP, Upland, CA, USA).

The positive samples were subjected to PCR again for species determination with the primers in Table 2. PCR cycles were 94 °C 2 min, 35 cycles (94°C 1 min, 55 °C 30 s, 72 °C 30 s), followed by 72 °C 5 min for *Mycoplasma hemofelis, Candidatus* Mycoplasma hemominutum, and *Candidatus* Mycoplasma turicensis. PCR products were run on 1.5% agarose gel electrophoresis and visualized in a UV transimulator (UVP, Upland, CA, USA).

### 2.6. Sequence Analysis of Mycoplasma spp. 16S rRNA Gene

FinchTV 1.4.0 (Geospiza Inc., Seattle, Washington, DC, USA) was used to visualize the sequence data. Sequence ends were trimmed by comparison with published sequences using the Basic Local Alignment Search Tool (BLAST) (URL https://blast.ncbi.nlm.nih.gov/Blast.cgi, accessed on 6 June 2024). The trimmed sequences were then transferred to the MEGA X program [28]. The alignment was performed using published reference sequences, and some other sequences retrieved from NCBI PubMed were included as outgroups. The most appropriate phylogenetic tree model was determined by the maximum likelihood method in the MEGA X program [28], and the tree was created. The tree was constructed using MEGA version X and the Hasegawa–Kishino–Yano (HKY) model. The numbers at the nodes indicate the percentage of occurrence of the clades in 1000 bootstrap replicates of the data.

### 2.7. Statistical Analysis

A cross-tabulation evaluation was conducted using categorical data, numbers, and percentages. In cases where the expected cells were less than 20%, the data were analyzed using the Monte Carlo simulation method. The data were analyzed using the SPSS 25 statistical package program (IBM Corp. Released 2017. IBM SPSS Statistics for Windows, Version 25.0. Armonk, NY, USA: IBM Corp.). Pearson Chi-square *p*-values were calculated to determine the statistical significance of the relationship among cat breed, age, gender, ownership status, and distribution of *Mycoplasma* spp. according to the districts of Konya. A *p*-value of less than 0.05 was considered statistically significant.

## 3. Results

### 3.1. Microscopy and PCR

No *Mycoplasma* spp. was detected in any cat as a result of the microscopic examination of thin blood smears. *Mycoplasma* spp. DNA was detected in 36 (9.4%) of 384 cats by PCR screening of genomic DNA samples with universal primers (HBT-F/HBT-R). All samples found to be positive by genus-specific PCR were subjected to repeat PCR with primers specific for feline *Mycoplasma* species. Species-specific PCR results were not obtained in some samples that were found to be positive with universal primer. For this reason, some of both HBT-PCR (n: 6) and species-specific PCR products (n: 2) were unidirectionally sequenced by a commercial company (BM Lab., Ankara, Turkey).

### 3.2. Phylogenetic Analysis

In the study, some amplicons obtained as a result of PCR analyses with universal primers and species-specific primers were sent to a commercial company for unidirectional sequence analysis. For this purpose, 15 PCR products were sent for sequence analysis, but good results were not obtained from all of them. Sequence analyses were requested several times in order to obtain as many sequences as possible within the project budget. As a result, eight *Mycoplasma* spp. sequences were obtained in a quality that can be uploaded to GenBank (National Center for Biotechnology Information). The sequences obtained in the study have been registered in GenBank under the following accession numbers: PP894221 for *M. hemofelis*; PP894220 for *Candidatus* Mycoplasma turicensis; PP889389, PP889390, PP889391, and PP889392 for *Candidatus* Mycoplasma hemominutum; PP800760 and PP800761 for *M. hemocanis*.

**Figure 2:**
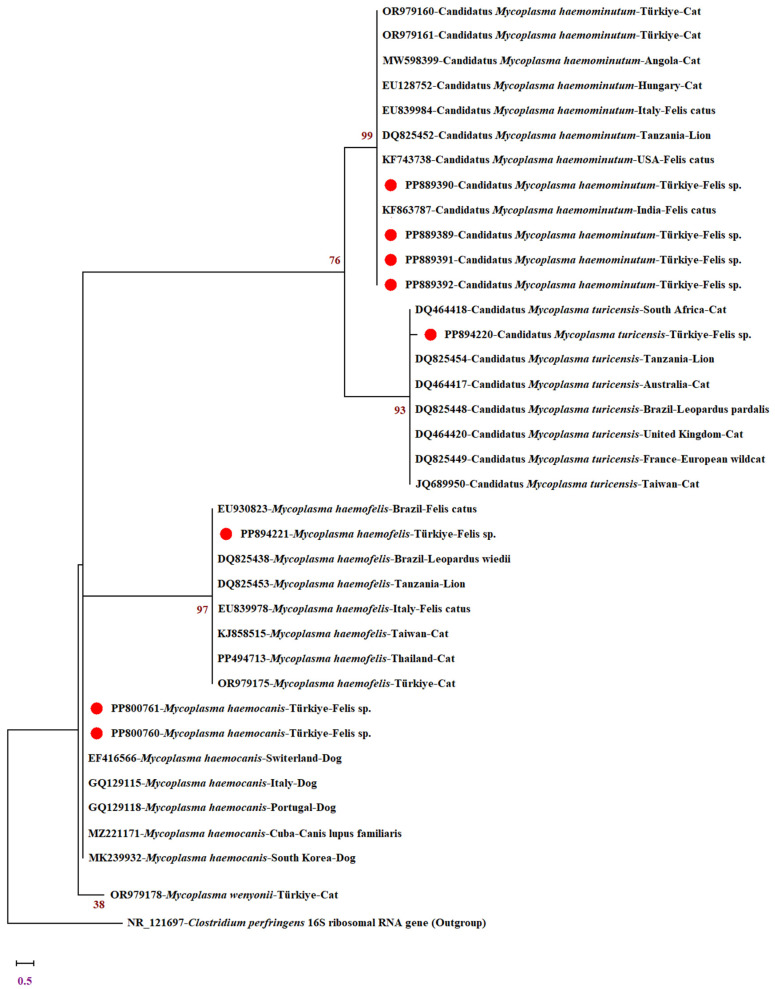
Maximum likelihood phylogram inferred from *Mycoplasma* sp. *16S rRNA* gene. The phylogenetic tree was constructed with the MEGA X version using Hasegawa–Kishino–Yano (HKY). Numbers in the nodes indicate the percentage of clades occurring in 1000 bootstrap replicates of the data. The *16S rRNA* sequences of *Mycoplasma* species in this study are shown with red dots. The *16S rRNA* gene sequence of *Clostridium perfringens* (NR121697) was used as an outgroup.

It was noteworthy that all *Mycoplasma* sp. isolates obtained in the study formed monophyletic groups with various feline hemotropic *Mycoplasma* species obtained from a study in Türkiye and different countries. The *M. hemofelis* isolate (PP894221) identified in the study formed a clade with domestic and wild feline *M. hemofelis* isolates from Brazil (EU930823, DQ825438), Italy (EU839978), Tanzania (DQ825453), Taiwan (KJ858515), Thailand (PP494713), and Türkiye (OR979175) with high nucleotide sequence similarity (99.26–99.80%). *M. hemocanis* isolates (PP800760, PP800761) formed a well-supported cluster with domestic canine *M. hemocanis* isolates from Italy (GQ129115), South Korea (MK239932), Cuba (MZ221171), Portugal (GQ129118) and Switzerland (EF416566) with a nucleotide sequence identity of 97.80–99.64%. The *Candidatus* Mycoplasma turicensis isolate was clustered with domestic and wild cat isolates from Australia (DQ464417), Brazil (DQ825448), France (DQ825449), South Africa (DQ464418), UK (DQ464420), Tanzania (DQ825454) and Taiwan (JQ689950). Finally, *C*Mhm isolates (PP889389, PP889390, PP889391, PP889392), the most common species in this study, were found to form clades with high nucleotide sequence similarity (98.83–100%) with isolates belonging to the same species from Angola (MW598399), America (KF743738), Italy (EU839984), India (KF863787), Hungary (EU128752), Tanzania (DQ825452), and Türkiye (OR979160, OR979161). The phylogenetic tree constructed with the sequences obtained in the study is presented in Figure 2.

### 3.3. Risk Factor Analysis

The findings obtained in the study were also statistically evaluated in terms of some parameters. Although no statistically significant relationship was found between the molecular prevalence of *Mycoplasma* sp. in cats and the sampled districts, breed, and gender of the cats (*p* > 0.05), it was concluded that the age of the cats and whether they were owned or not were effective on the distribution of feline hemotropic mycoplasmosis infection (*p* < 0.05). Detailed information about the statistical findings is given in Table 3.

## 4. Discussion

Given the inadequacy of microscopic diagnosis of feline hemotropic mycoplasmosis, especially at low levels of bacteriemia, the need for molecular methods is growing every day [29]. Indeed, the fact that *Mycoplasma* species with epierythrocytic localization were not found in Giemsa-stained thin blood smears but were positive by PCR in some of the same samples, supports this situation. *Mycoplasma* spp. were not detected microscopically in any of the cats examined in the study, and a molecular prevalence of 9.4% (n: 36) was determined by PCR on the same materials. This once again points to the superiority of molecular techniques over microscopic diagnosis. PCR-based techniques provide reliable results even at low levels of parasitemia. Molecular techniques have a significant role in identifying animals that are carriers of infection, especially where parasitemia is low, and it is recommended that pathogens such as mycoplasmas, which are challenging to diagnose microscopically, be confirmed by molecular techniques in order not to miss infections [30].

Feline hemotropic mycoplasmosis has been reported to cause mortality in domestic cats in Türkiye [18,31], and molecular prevalence was high in cats suspected of having the disease [20,21]. Studies on the subject show that the number of studies on the molecular epidemiology of the disease in cats from Türkiye is constricted. In some of these studies, feline hemoplasma species have been molecularly identified in Antalya (17.6%), Ankara (23.1%), Bursa (7.7%), İstanbul (8.54–19.3%), İzmir (17.5%), Kayseri (9.5%), and Tekirdağ (11.4%) provinces [16,21,32,33,34]. In a comprehensive epidemiological study recently conducted by Ceylan et al. [6], the molecular prevalence of feline hemotropic mycoplasmosis was determined as 11.2% in cats sampled from many provinces of Türkiye. *Candidatus* Mycoplasma hemominutum was reported as the main causative species of infections in most of these studies [18,20,21], while in some studies, the species could not be identified [34]. The molecular analysis of feline hemotropic mycoplasma species across Türkiye revealed three distinct *Mycoplasma* species: *Mhf*, *C*Mt, and *C*Mhm. Cetinkaya et al. [16] determined the molecular prevalences to be 9.9%, 0.8%, and 17.7%, respectively, for each species in Istanbul. While some regional studies have been carried out, a comprehensive understanding of the molecular phylogenetic characterization of the identified *Mycoplasma* species remains elusive in Türkiye [6,16,34]. This study comprehensively revealed *Mycoplasma* spp.’s molecular prevalence in the Konya province of Türkiye and evaluated the prevalence of infection in terms of some risk factors. The *16S rRNA* gene fragment-based genus-specific PCR assay showed a 9.4% *Mycoplasma* spp. molecular prevalence in the feline blood samples representing Konya province and its districts. The study results also showed that *Mycoplasma* spp. infection was statistically more prevalent in stray cats and cats over one year of age. This may be attributed to the increased probability of exposure to vectors with age, regular ectoparasite control in owned cats, and less interaction with the external environment compared to stray cats. There are studies investigating various risk factors for feline hemotropic mycoplasmosis [33,35,36,37,38], some of which showed a statistically significant relationship between feline hemoplasma prevalence and age [38] and ownership status [33], which supports the findings of this study. In contrast to this situation, although there are studies showing that the prevalence of infection is more common in male cats due to behavioral characteristics such as fighting and roaming, which may increase the exposure of cats to arthropod vectors and other infected cats [6,29,39], no statistically significant relationship was found between the prevalence of infection and the sex of cats in this study (*p* = 0.601). This may be attributed to the fact that most of the cat population studied are owned cats and live in isolated areas from the external environment.

Direct sequencing of some PCR products of positive *Mycoplasma* spp. samples and species-specific PCR products confirmed that four samples belonged to *C*Mhm, two to *M. hemocanis*, one to *M. hemofelis*, and one to *C*Mt. The current study has revealed the presence of various *Mycoplasma* species, including *Mhf*, *C*Mhm, *Mhc*, and *C*Mt, affecting cats in Türkiye, highlighting the further understanding of their clinical and epidemiological importance. The study findings demonstrated that *C*Mhm is the primary species involved in feline hemoplasmosis, which is in agreement with some previous studies in Türkiye [6,18,20,21,33]. There are also molecular epidemiological studies conducted in different countries to support this finding [38,40,41,42]. As one of the study’s notable findings, *M. hemocanis* DNA detection in cats for the first time in Konya province (Selçuklu and Altınekin districts) in Türkiye indicates that further studies should be carried out on the vector arthropods involved in the transmission, the transmission dynamics of feline hemoplasma species, and whether this species is essential in terms of clinical infection in hosts other than its original host. In a recent study, the DNA of pathogens not belonging to the felids was detected in cats [6]. Ceylan et al. [6] detected *Mycoplasma wenyonii*, which is a pathogenic hemoplasma species for cattle, and *Babesia ovis* DNA, which is the primary etiological agent of ovine babesiosis, in cat blood samples from many provinces of Türkiye and confirmed the results by sequence analysis. In Türkiye, there is some literature indicating that nucleic acids of various pathogens can be detected in non-specific hosts other than cats [43,44]. The detection of *M. hemocanis* in cats also emphasizes the potential for inter-species transmission and underlines the complex interactions within the ecosystem that facilitate the spread of FVBDs. In addition to vector-borne transmission, interspecific interactions such as scratching, biting, and hunting are considered to have a role in the detection of DNA of some pathogens in unspecific hosts. As far as the authors are aware, the present study is also one of the most broad-reaching studies regarding the phylogeny of feline hemotropic *Mycoplasma* species in Türkiye [6] and shows that the cat-specific hemoplasmas cluster together with isolates registered from various countries with high sequence identities. It was determined that *M. hemofelis*, *C*Mhm, and *C*Mt isolates detected in the study formed monophyletic groups with *Mycoplasma* isolates reported from domestic and wild cats from different countries. This corroborates studies indicating a worldwide distribution of *Mycoplasma* species with no major phylogeographic differences between regions and species. On the other hand, *M. hemocanis* isolates (PP800760, PP800761), which have been reported in cats for the first time, clustered with domestic and wild canine isolates reported from Italy (GQ129115), South Korea (MK239932), Cuba (MZ221171), Portugal (GQ129118), and Switzerland (EF416566) with 97.80–99.64% nucleotide sequence similarity. This highlights the complex nature of the disease and points to the need for further research to unravel the clinical significance and transmission dynamics of *M. hemocanis* infections in cats.

## 5. Conclusions

In conclusion, this comprehensive molecular prevalence study in domestic cats of Konya province in Türkiye showed that four infective hemotropic *Mycoplasma* species in cats are circulating in different districts of the city, indicating that it is a neglected health problem for cats and public health. Given the impact of such zoonotic pathogens on “One Health”, it is recommended that routine screening, public awareness raising, effective control, and prophylactic strategies be implemented to minimize infection in cats and, subsequently, in humans. To prevent the spread of hemotropic mycoplasmosis, it is advised that donor animals be molecularly screened for *Mycoplasma* species before blood transfusions. Additionally, the routine treatment of cats with insecticides or acaricides should be implemented to protect them from arthropod vectors. While keeping cats indoors to reduce their interaction with vectors may not always be practical, it can help minimize exposure. Currently, no commercial vaccine is available to protect cats from hemotropic mycoplasmosis, making these preventive measures even more critical for safeguarding both feline and human health due to the zoonotic potential of certain hemoplasma species.

## Figures and Tables

**Table 1:** Information about the cats included in the study.

Districts	n	Breed (n)	Gender	Age	Status
Ahırlı	3	Crossbreed (1), Tabby (1), Scottish Fold (1)	1 ♀ 2 ♂	<1 y: 2 >1 y: 1	3 owned
Akören	2	Tabby (2)	1 ♀ 1 ♂	<1 y: 0 >1 y: 2	2 owned
Akşehir	2	Crossbreed (1), Exotic Shorthair (1)	1 ♀ 1 ♂	<1 y: 0 >1 y: 2	2 owned
Altınekin	2	Crossbreed (1), Tabby (1)	1 ♀ 1 ♂	<1 y: 0 >1 y: 2	2 owned
Beyşehir	6	British Shorthair (1), Crossbreed (1), Orange Tabby (1), Scottish Fold (1), Tabby (2)	1 ♀ 5 ♂	<1 y: 1 >1 y: 5	6 owned
Bozkır	6	British Shorthair (2), Crossbreed (1), Tabby (3)	2 ♀ 4 ♂	<1 y: 0 >1 y: 6	6 owned
Cihanbeyli	4	British Shorthair (2), Crossbreed (2)	1 ♀ 3 ♂	<1 y: 0 >1 y: 4	4 owned
Çumra	13	British Shorthair (1), Crossbreed (6), Orange Tabby (1), Persian (1), Scottish Fold (1), Scottish Straight (1), Tabby (1), Van (1)	5 ♀ 8 ♂	<1 y: 5 >1 y: 8	13 owned
Doğanhisar	4	British Shorthair (1), Crossbreed (2), Persian (1)	4 ♀	<1 y: 1 >1 y: 3	4 owned
Ereğli	9	British Shorthair (2), Crossbreed (1), Orange Tabby (1), Tabby (5),	3 ♀ 6 ♂	<1 y: 2 >1 y: 7	9 owned
Güneysınır	1	Tabby (1)	1 ♂	<1 y: 0 >1 y: 1	1 owned
Hadim	4	Ankara (1), Crossbreed (2), Tabby (1)	3 ♀ 1 ♂	<1 y: 1 >1 y: 3	4 owned
Halkapınar	1	Crossbreed (1)	1 ♀	<1 y: 0 >1 y: 1	1 owned
Ilgın	13	Crossbreed (3), Norwegian Forest Cat (1), Tabby (9)	5 ♀ 8 ♂	<1 y: 1 >1 y: 12	13 owned
Kadınhanı	10	Ankara (2), British Shorthair (1), Crossbreed (3), Persian (1), Tabby (2), Van (1)	3 ♀ 7 ♂	<1 y: 2 >1 y: 8	10 owned
Karapınar	4	British Shorthair (1), Crossbreed (1), Tabby (2)	2 ♀ 2 ♂	<1 y: 1 >1 y: 3	4 owned
Karatay	64	Ankara (2), British Longhair (3), British Shorthair (4), Crossbreed (25), Maine Coon (1), Orange Tabby (2), Persian (1), Scottish Fold (4), Smokin (1), Tabby (19), Van (2)	31 ♀ 33 ♂	<1 y: 11 >1 y: 53	64 owned
Kulu	2	Scottish Fold (2)	2 ♂	<1 y: 0 >1 y: 2	2 owned
Meram	65	Ankara (1), Bombay (1), British Shorthair (5), Crossbreed (17), Orange Tabby (1), Russian Blue (2), Scottish Fold (5), Scottish Shorthair (1), Scottish Straight (2), Somali (1), Tabby (27), Tuxedo (1), Van (1)	30 ♀ 35 ♂	<1 y: 7 >1 y: 58	65 owned
Sarayönü	6	American Bobtail (1), Crossbreed (1), Scottish Fold (1), Tabby (3)	3 ♀ 3 ♂	<1 y: 1 >1 y: 5	6 owned
Selçuklu	149	Ankara (6), Bombay (1), British Shorthair (6), Crossbreed (102), Exotic Shorthair (1), Persian (2), Scottish Fold (3), Siamese (1), Tabby (26), Van (1)	81 ♀ 68 ♂	<1 y: 20 >1 y: 129	65 owned 84 stray
Seydişehir	9	British Shorthair (1), Crossbreed (4), Scottish Fold (1), Tabby (3)	6 ♀ 3 ♂	<1 y: 1 >1 y: 8	9 owned
Taşkent	5	Crossbreed (1), Tabby (4)	5 ♀	<1 y: 1 >1 y: 4	5 owned
Total	384	American Bobtail (1), Ankara (12), Bombay (2), British Longhair (3), British Shorthair (27), Exotic Shorthair (2), Persian (6), Maine Coon (1), Crossbreed (176), Norwegian Forest Cat (1), Russian Blue (2), Orange Tabby (6), Scottish Fold (19), Scottish Shorthair (1), Scottish Straight (3), Siamese (1), Smokin (1), Somali (1), Tabby (112), Tuxedo (1), Van (6)	190 ♀ 194 ♂	<1 y: 57 >1 y: 327	300 owned 84 stray

**Table 2:** Information concerning the primers used in PCR analyses.

Mycoplasma Spesies	Primer Sequence	Target Gene	Bp	References
*Mycoplasma* spp.	HBT-F, ATA CGG CCC ATA TTC CTA CG HBT-R, TGC TCC ACC ACT TGT TCA	*16s rRNA*	595 bp	[24]
*Mycoplasma hemofelis*	Hf-F, 5′-ACG AAA GTC TGA TGG AGC AAT A-3′ Hf-R, 5′-ACG CCC AAT AAA TCC GRA TAA T-3′	*16s rRNA*	170 bp	[25]
*Candidatus* Mycoplasma hemominutum	Hf-F, 5′-ACG AAA GTC TGA TGG AGC AAT A-3′ Hf-R, 5′-ACG CCC AAT AAA TCC GRA TAA T-3′	*16s rRNA*	193 bp	[25]
*Candidatus* Mycoplasma turicensis	Mt1-F, 5′-GTATCCTCCATCAGACAGAA-3′ Mt2-R, 5′-CGCTCCATATTTAATTCCAA-3′	*16s rRNA*	488 bp	[26]
*Mycoplasma hemocanis*	Mhcf,5′-GAAACTAAGGCCATAAATGACGC-3′ Mhcr, 5′-ACCTGTCACCTCGATAACCTCTAC-3′	*16s rRNA*	309 bp	[27]
*Candidatus Mycoplasma hematoparvum*	CMhpf, 5′-ACGAAAGTCTGATGGAGCAATAC-3′ CMhpr, 5′-TATCTACGCATTCCACCGCTAC-3′	*16s rRNA*	328 bp	[27]

**Table 3:** Statistical analysis results.

		*Mycoplasma* sp. Positive (n)	*Mycoplasma* sp. Negative (n)	
**Districts**	Ahırlı (n: 3)	0	3	*p* = 0.322 χ^2^ = 25.112
	Akören (n: 2)	0	2	
	Akşehir (n: 2)	0	2	
	Altınekin (n: 2)	1	1	
	Beyşehir (n: 6)	0	6	
	Bozkır (n: 6)	1	5	
	Cihanbeyli (n: 4)	0	4	
	Çumra (n: 13)	0	13	
	Doğanhisar (n: 4)	0	4	
	Ereğli (n: 9)	0	9	
	Güneysınır (n: 1)	0	1	
	Hadim (n: 4)	1	3	
	Halkapınar (n: 1)	0	1	
	Ilgın (n: 13)	4	9	
	Kadınhanı (n: 10)	0	10	
	Karapınar (n: 4)	0	4	
	Karatay (n: 64)	7	57	
	Kulu (n: 2)	0	2	
	Meram (n: 65)	2	63	
	Sarayönü (n: 6)	0	6	
	Selçuklu (n: 149)	19	130	
	Seydişehir (n: 9)	1	8	
	Taşkent (n: 5)	0	5	
**Breed**	American Bobtail (n: 1)	0	1	*p* = 0.302 χ^2^ = 20.289
	Ankara (n: 12)	1	11	
	Bombay (n: 2)	0	2	
	British Longhair (n: 3)	0	3	
	British Shorthair (n: 27)	2	25	
	Crossbreed (n: 176)	27	149	
	Exotic Shorthair (n: 2)	0	2	
	Maine Coon (n: 1)	0	1	
	Norwegian Forest Cat (n: 1)	0	1	
	Persian (n: 6)	0	6	
	Russian Blue (n: 2)	1	1	
	Sarman (n: 6)	0	6	
	Scottish Fold (n: 20)	0	20	
	Scottish Shorthair (n: 3)	0	3	
	Scottish Straight (n: 1)	0	1	
	Siamese (n: 1)	0	1	
	Smokin (n: 1)	0	1	
	Somali (n: 1)	0	1	
	Tuxedo (n: 1)	0	1	
	Tabby (n: 111)	5	106	
	Van (n: 7)	0	7	
**Gender**	Female (n: 190)	16	174	*p* = 0.601 χ^2^ = 0.403
	Male (n: 194)	20	174	
**Age**	>1 year (n: 57)	0	57	*p* = 0.005 χ^2^ = 6.924
	<1 year (n: 327)	36	291	
**Status**	Owned (n: 300) Stray (n: 84)	19 17	281 67	*p* = 0.000 χ^2^ = 14.934

## Data Availability

The datasets generated and/or analyzed during the present study are available in the National Center for Biotechnology Information GenBank database (URL https://www.ncbi.nlm.nih.gov/, accessed on 15 June 2024) under the accession numbers: PP894220, PP894221, PP889389, PP889390, PP889391, PP889392, PP800760 and PP800761.
